# Diurnal and stress‐induced intra‐hippocampal corticosterone rise attenuated in 11β‐HSD1‐deficient mice: a microdialysis study in young and aged mice

**DOI:** 10.1111/ejn.12836

**Published:** 2015-01-23

**Authors:** Joyce L. W. Yau, June Noble, Christopher J. Kenyon, Mike Ludwig, Jonathan R. Seckl

**Affiliations:** ^1^ Centre for Cognitive Aging and Cognitive EpidemiologyUniversity of EdinburghEdinburghUK; ^2^ Endocrinology UnitBHF Centre for Cardiovascular Science, Queen's Medical Research InstituteUniversity of Edinburgh47 Little France CrescentEdinburgh EH16 4TJUK; ^3^ Centre for Integrative PhysiologyUniversity of EdinburghEdinburghUK

**Keywords:** ageing, glucocorticoids, hippocampus, hypothalamic–pituitary–adrenal axis

## Abstract

11β‐Hydroxysteroid dehydrogenase type 1 (11β‐HSD1) locally regenerates active glucocorticoids from their inert forms thereby amplifying intracellular levels within target tissues including the brain. We previously showed greater increases in intra‐hippocampal corticosterone (CORT) levels upon Y‐maze testing in aged wild‐type than in 11β‐HSD1^−/−^ mice coinciding with impaired and intact spatial memory, respectively. Here we examined whether ageing influences 11β‐HSD1 regulation of CORT in the dorsal hippocampus under basal conditions during the diurnal cycle and following stress. Intra‐hippocampal CORT levels measured by *in vivo* microdialysis in freely behaving wild‐type mice displayed a diurnal variation with peak levels in the evening that were significantly elevated with ageing. In contrast, the diurnal rise in intra‐hippocampal CORT levels was greatly diminished in 11β‐HSD1^−/−^ mice and there was no rise with ageing; basal intra‐hippocampal CORT levels were similar to wild‐type controls. Furthermore, a short (3 min) swim stress induced a longer lasting increase in intra‐hippocampal CORT levels in wild‐type mice than in 11β‐HSD1^−/−^ mice despite no genotypic differences in elevation of plasma CORT. These data indicate that 11β‐HSD1 activity contributes substantially to diurnal and stress‐induced increases in hippocampal CORT levels. This contribution is even greater with ageing. Thus, 11β‐HSD1 inhibition may be an attractive target for treating cognitive impairments associated with stress or ageing.

## Introduction

Glucocorticoids are potent modulators of mood and memory (De Kloet *et al*., 1998; de Quervain *et al*., 2009). The rodent glucocorticoid, corticosterone (CORT), is synthesised in the adrenal cortex and released into the blood upon activation of the hypothalamic–pituitary–adrenal (HPA) axis. CORT circulates mostly bound to corticosteroid‐binding globulin (CBG), with the remaining free CORT fraction readily reaching the brain where it binds to intracellular receptors, the high‐affinity mineralocorticoid receptors and lower affinity glucocorticoid receptors (GRs; Herman *et al*., 1989; de Kloet *et al*., 1993). Both receptors are abundant in limbic regions of the brain, particularly in the hippocampus, a structure known to play a critical role in memory (McEwen & Sapolsky, 1995; Barbosa *et al*., 2012) and in the HPA axis stress response (Herman *et al*., 2005). Within specific cells in the brain, including the hippocampus, 11β‐hydroxysteroid dehydrogenase type 1 (11β‐HSD1) catalyses the local regeneration of CORT from its inert 11keto derivative, 11‐dehydrocorticosterone (Wyrwoll *et al*., 2011), thereby amplifying intracellular CORT levels.

While elevated circulating CORT levels associated with ageing have been widely shown to impair hippocampus‐dependent spatial memory in rodents (Issa *et al*., 1990; Yau *et al*., 1995; Vallee *et al*., 1999), our studies support a central role of 11β‐HSD1‐generated CORT in this process (Yau & Seckl, 2012). Thus, 11β‐HSD1^−/−^ mice failed to show the impaired spatial memory found in aged wild‐type mice despite similar age‐related elevations in plasma CORT levels (Yau *et al*., 2001, 2007). Recently, we found that intra‐hippocampal CORT levels rise rapidly during the acquisition and retrieval trials in the Y‐maze spatial memory task, and that it is the 11β‐HSD1‐generated CORT from this rise during retrieval that associates with impaired memory in aged wild‐type mice (Yau *et al*., 2015). This suggests a dynamic regulation of intracellular CORT by 11β‐HSD1 as required during periods of enhanced HPA axis activity.

Under basal conditions, circulating CORT levels are highly variable, undergoing both diurnal and approximate hourly ultradian pulses during the peak active phase (de Kloet & Sarabdjitsingh, 2008; Spiga *et al*., 2014). These normal daily variations in glucocorticoid secretion appear important for the maintenance of physiological functions. Disruption of the normal secretion pattern is associated with vulnerability to stress‐related diseases, such as depression (Young *et al*., 2004). Basal morning plasma CORT levels tend to be elevated with ageing in rodents (Issa *et al*., 1990; Yau & Seckl, 2012), although this can vary depending on study design (Sapolsky, 1991). Whether ageing affects the diurnal variation of CORT levels in murine plasma or the brain is unknown. In the present study we investigated the effects of ageing on 11β‐HSD1‐generated CORT in the dorsal hippocampus during the diurnal rhythm and following acute swim stress using an *in vivo* microdialysis approach in freely behaving wild‐type and 11β‐HSD1^−/−^ mice.

## Materials and methods

### Animals

Male mice homozygous for targeted disruption of the 11β‐HSD1 gene, congenic on the C57BL/6J genetic background (Kotelevtsev *et al*., 1997; Carter *et al*., 2009), and wild‐type control mice were bred and maintained within our biomedical research facility housed under standard conditions on a 12 h light/dark cycle (lights on at 07:00 h), with food and water *ad libitum* until experimentation at either 6 months (young) or 24 months (aged). All animal procedures were approved by the local University of Edinburgh animal welfare ethical review body and performed in strict accordance with the UK Animals (Scientific Procedures) Act, 1986.

### Surgery

All mice were anesthetised with isoflurane. With the head fixed in a stereotaxic frame (David Kopf), a microdialysis guide cannula (CMA/7, CMA Microdialysis, Sweden) was implanted to just enter the dorsal hippocampus [coordinates relative to bregma – anteroposterior −2.3 mm; lateral + 1.5 mm (midline); dorsoventral −1.5 mm from skull surface (Paxinos & Franklin, 2003)]. During surgery, the body temperature was maintained with an electric thermally controlled blanket. Dental cement and two small anchor screws were used to secure the guide cannula and a small metal peg (for later connection to a liquid swivel) on the skull. The mice were allowed to recover for 7 days before the microdialysis procedure.

### Microdialysis procedure

Under isoflurane anesthesia, a dialysis probe (CMA/7; CMA Sweden; length –1 mm; molecular cut‐off – 6 kDa; and membrane outer diameter –0.24 mm) was lowered through the pre‐implanted guide cannula so that the 1‐mm microdialysis membrane was exposed to the inside of the CA1 dorsal hippocampus, a subregion known to play an important role in learning and memory (Rempel‐Clower *et al*., 1996; Kesner *et al*., 2005; Lee *et al*., 2005; Vago *et al*., 2007; Dillon *et al*., 2008).

Mice were individually housed in a clear round‐bottom bowl system to allow free movement (CMA/120, Sweden) and constant access to water and food. The probe was perfused continuously overnight with sterile artificial cerebrospinal fluid at 0.3 μL/min using a microinfusion pump to equilibrate extracellular metabolites and to allow the animal to acclimatise to the liquid swivel counterbalance arm system. Fluorethylenepolymer tubing with a dead volume of 1.2 μL/100 mm length was used for the connections. Microdialysis samples were collected in cooled plastic vials using an automated refrigerated fraction collector (CMA 470, Sweden).

### Experiment 1 – diurnal intra‐hippocampal CORT levels in freely behaving wild‐type and 11β‐HSD1^−/−^ mice

Morning (08:00–10:00 h) tail venesection blood samples were first taken for measurement of basal CORT levels in young (6 months) [wild‐type (*n* = 7) and 11β‐HSD1^−/−^ (*n* = 6)] mice and aged (24 months) [wild‐type (*n* = 6) and 11β‐HSD1^−/−^ (*n* = 6)] mice. A few days later, mice underwent surgery for implantation of the guide cannula to allow insertion of the microdialysis probe on the day of the experiment (see above). On the morning following overnight acclimatisation, dialysate samples were collected at 0.3 μL/min flow rate every hour from 12:00 to 09:00 h over 2 days in parallel from two age‐ and genotype‐matched mice.

Diurnal plasma CORT levels were measured in a separate cohort of aged (24 months) wild‐type (*n* = 9) and 11β‐HSD1^−/−^ (*n* = 9) mice from tail venesection blood samples taken at 07:00, 14:00, 17:00 and 19:00 h. The tail nick blood samples were collected from each mouse over a period of 2 days (two time points per day).

### Experiment 2 – intra‐hippocampal CORT levels during acute stress in freely behaving wild‐type and 11β‐HSD1^−/−^ mice

Young and aged mice [young wild‐type (*n* = 5) and 11β‐HSD1^−/−^ (*n* = 4); aged wild‐type (*n* = 8) and 11β‐HSD1^−/−^ (*n* = 7)] were prepared for *in vivo* microdialysis as described above. After collecting consecutive 10‐min baseline intra‐hippocampal samples for 1 h, each mouse was subjected to a tail nick blood sample (basal) followed immediately by a 3‐min forced swim in a clear plastic beaker (27 cm high, 22 cm diameter, 25 °C with water filled to a level that prevented paws and tail from touching the bottom). Further tail nick blood samples were collected at the end of the swim stress and 3 h later.

Mice were killed at the end of each microdialysis experiment by cervical dislocation, and brains were removed and frozen on powdered dry ice. Correct hippocampal probe placement was verified histologically in 30‐μm cryostat sections stained with pyronin, and only data from mice with correct probe placement were included for analysis (two young and one aged wild‐type, and one aged 11β‐HSD1^−/−^ mice were omitted). Microdialysis samples were stored at −80 °C for later determination of CORT concentrations.

### CORT

Plasma CORT levels were measured using an in‐house radioimmunoassay (RIA) with [^3^H]‐CORT (Yau *et al*., 2011). Intra‐hippocampal CORT levels were measured in 10‐μL dialysate samples using a more sensitive RIA with [^125^I]‐CORT to detect the much lower brain CORT levels. The intra‐assay coefficient of variation was 4% and detection limit approximately 1.4 fmoles/sample.

### Data analysis

All data were analysed using graphpad prism 6 software. Diurnal and stress hippocampal and plasma CORT data in young and aged mice were analysed using two‐way anova with time and genotype or time and age as independent variables, followed by *post hoc* Bonferroni's Multiple Comparison tests for individual between‐group comparisons as appropriate. The areas under the curve (AUCs; arbitrary units) for stress hippocampal CORT data were compared between genotypes by unpaired *t*‐test. Significance was set at *P* < 0.05. Data are expressed as mean ± SEM.

## Results

### Effect of 11β‐HSD1 deficiency on diurnal intra‐hippocampal CORT levels in young and aged mice

**Figure 1:**
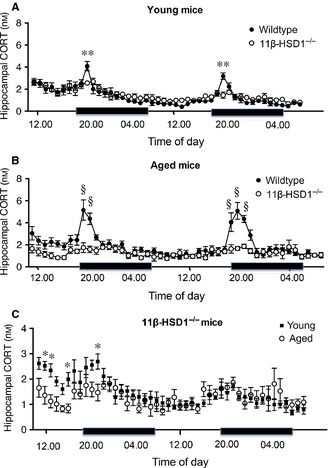
Effect of 11β‐hydroxysteroid dehydrogenase type 1 (11β‐HSD1) deficiency on the diurnal rhythm of hippocampal corticosterone (CORT) levels in freely behaving young and aged mice. (A, B) Intra‐hippocampal CORT levels sampled every hour by *in vivo* microdialysis over 2 days in young 6‐month‐old and aged 24‐month‐old wild‐type and 11β‐HSD1^−/−^ mice. **P < 0.01, §*P* < 0.0001 vs. corresponding time point in 11β‐HSD1^−/−^ mice, *n* = 6–7 per genotype. (C) Highlights data for young and aged 11β‐HSD1^−/−^ mice within a narrower range of hippocampal CORT values. *P < 0.05 vs. corresponding time point in aged 11β‐HSD1^−/−^ mice. Black bar represents the dark period. Means ± SEM are shown.

Under basal conditions, morning (07:00–12:00 h) intra‐hippocampal CORT levels measured in freely behaving young and aged mice were not affected by 11β‐HSD1 deficiency (Fig. 1A and B). In normal mice, CORT release is increased in the evening in response to diurnal cues. anova of the intra‐hippocampal CORT levels in wild‐type and 11β‐HSD1^−/−^ mice showed a significant effect of time of day (young, *F*_45,506_ = 13.5, *P* < 0.0001; aged, *F*_45,460_ = 7.6, *P* < 0.0001), genotype (young, *F*_1,506_ = 6.0, *P* < 0.05; aged, *F*_1,460_ = 89.2, *P* < 0.0001) and genotype × time interaction (young, *F*_45,506_ = 1.7, *P* < 0.01; aged, *F*_45,460_ = 4.0, *P* < 0.0001; Fig. 1A and B). Hourly dialysate samples from the dorsal hippocampus collected over 2 days in freely behaving mice revealed clear diurnal rhythms of intra‐hippocampal CORT in young and aged wild‐type mice, with peak values 1 h into the onset of the dark period at 20:00 h in young and aged mice (Fig. 1A and B). The increase was greater and lasted longer in aged (peak rise 2.5‐fold of baseline values, increased from 19.00 to 21:00 h) compared with young (peak rise 1.5‐fold of baseline values, increased at 20:00 h only) wild‐type mice (*F*_1,506_ = 84.4, *P* < 0.0001; Fig. 1B). In contrast, intra‐hippocampal CORT levels in 11β‐HSD1^−/−^ mice were lower than age‐matched wild‐type mice and did not show a clear diurnal rhythm (Fig. 1A and B). The nocturnal increases in intra‐hippocampal CORT levels were barely detectable when compared with wild‐type mice, but were significant in young [1.70 ± 0.14 nm (21:00 h) vs. 1.05 ± 0.06 nm mean baseline morning (07:00–12:00 h) values, 62% rise, *P* < 0.01, *t*‐test] and aged 11β‐HSD1^−/−^ mice [1.67 ± 0.17 nm (20:00 h) and 1.86 ± 0.21 nm (21:00 h) vs. 0.99 ± 0.07 nm mean baseline morning (07:00–12:00 h) values, 69–88% rise, *P* < 0.01, *t*‐tests; Fig. 1C]. Although age *per se* had no significant effect on intra‐hippocampal CORT levels in 11β‐HSD1^−/−^ mice, there was a significant effect of time of day (*F*_45,225_ = 5.3, *P* < 0.0001) and time × age interaction (*F*_45,225_ = 2.4, *P* < 0.0001; Fig. 1C). *Post hoc* analysis indicated that intra‐hippocampal CORT levels were significantly higher in young 11β‐HSD1^−/−^ mice (*P* < 0.05 compared with aged mice) at several time points during the first day of dialysate sampling, but not on day 2 when all mice were fully acclimatised to the microdialysis procedure (Fig. 1C).

**Figure 2:**
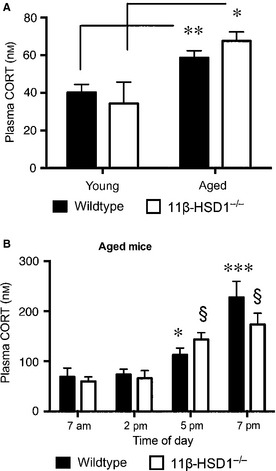
Diurnal plasma corticosterone (CORT) levels in wild‐type and 11β‐hydroxysteroid dehydrogenase type 1 (11β‐HSD1^−/−^) mice. (A) Basal morning plasma CORT levels in young (6 months) and aged (24 months) wild‐type and 11β‐HSD1^−/−^ mice (*n* = 6–7 per genotype) prior to the microdialysis collection of hippocampal CORT samples. **P < 0.01, *P < 0.05 vs. corresponding young mice. (B) Basal plasma CORT levels measured at different time points throughout the day in a separate cohort of aged (24 months) wild‐type (*n* = 9) and 11β‐HSD1^−/−^ mice (*n* = 9). *P < 0.05, ***P < 0.001, §*P* < 0.0001 compared with the corresponding 07:00 h time point CORT levels. Means ± SEM are shown.

In line with previous findings, plasma CORT levels were not significantly different in wild‐type and 11β‐HSD1^−/−^ mice, although basal (08:00–10:00 h) levels were higher in aged mice (*F*_1,20_ = 17.7, *P* < 0.001) of both genotypes (Fig. 2A). In a separate group of aged mice, diurnal changes in plasma CORT levels were measured, and showed that both wild‐type and 11β‐HSD1^−/−^ mice exhibited the expected nocturnal increases (*F*_3,72_ = 25.5, *P* < 0.0001; Fig. 2B) with no genotypic difference.

### Effect of 11β‐HSD1 deficiency on the stress‐induced rise in intra‐hippocampal CORT levels in young and aged mice

**Figure 3:**
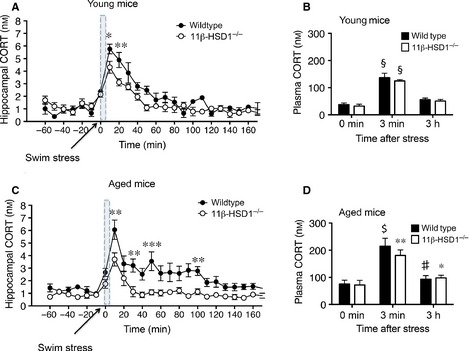
Acute swim stress induced rise in intra‐hippocampal corticosterone (CORT) levels in young and aged wild‐type and 11β‐hydroxysteroid dehydrogenase type 1 (11β‐HSD1^−/−^) mice. (A, B) Effect of stress (tail nick immediately before and after a 3‐min forced swim, shaded area) on intra‐hippocampal CORT and plasma CORT levels in young (6 months) wild‐type (*n* = 3) and 11β‐HSD1^−/−^ mice (*n* = 4). (A) *P < 0.05, **P < 0.01 compared with corresponding time point in 11β‐HSD1^−/−^ mice; (B) §*P* < 0.0001 compared with the corresponding 0 min or 3 h time points. (C, D) Effect of stress (tail nick immediately before and after a 3‐min forced swim, shaded area) on intra‐hippocampal CORT and plasma CORT levels in aged (24 months) wild‐type (*n* = 7) and 11β‐HSD1^−/−^ mice (*n* = 6). (C) **P < 0.01, ***P < 0.001 compared to corresponding time point in 11β‐HSD1^−/−^ mice; (D) $*P* < 0.0001, **P < 0.01 compared with the corresponding 0 min time point. ♯*P* < 0.001, *P < 0.05 compared with the corresponding 3 min time point. Means ± SEM are shown.

Acute stress (forced swim) significantly increased intra‐hippocampal CORT levels (*F*_23,109_ = 23.9, *P* < 0.0001) in young (6 month) mice, with levels peaking within 10 min and returning to baseline levels by 60 min in both genotypes (Fig. 3A). However, the peak response (*P* < 0.01) and AUC (wild‐type: 411 ± 14; 11β‐HSD1^−/−^: 322 ± 18, *t*‐test, *P* < 0.05) was greater in wild‐type than 11β‐HSD1^−/−^ mice (Fig. 3A). In contrast, there was no effect of genotype on plasma CORT responses to swim stress, which were increased more than twofold (*F*_2,15_ = 88.5, *P* < 0.0001; Fig. 3B), returning to baseline by the 3 h time point.

In aged mice, the swim stress also significantly increased intra‐hippocampal CORT levels (*F*_23,264_ = 10.52, *P* < 0.0001) within 10 min, with peak levels in wild‐type that were 63% greater than 11β‐HSD1^−/−^ mice (*P* < 0.01; Fig. 3C). This genotypic difference continued throughout the 3 h post‐stress period (*F*_1,11_ = 14.63, *P* < 0.01). CORT levels in aged 11β‐HSD1^−/−^ mice returned to basal values within 40 min, while wild‐type mice levels remained higher throughout the collection period (AUC, wild‐type: 522 ± 60 and 11β‐HSD1^−/−^: 248 ± 31; *t*‐test, *P* < 0.01; Fig. 3C). Plasma CORT levels in contrast showed no genotype effect in the aged mice increasing (more than twofold) following the swim stress and returning to basal levels at the 3 h time point (*F*_2,30_ = 22.4, *P* < 0.0001; Fig. 3D) in both groups.

Overall, increases in intra‐hippocampal CORT levels following the swim stress were not affected by age in either wild‐type or 11β‐HSD1^−/−^ mice (Fig. 3A and C). Plasma CORT levels were significantly higher after stress in aged wild‐type (*F*_1,24_ = 7.2, *P* < 0.05) and 11β‐HSD1^−/−^ mice (*F*_1,21_ = 30.45, *P* < 0.001) compared with young mice (Fig. 3B and D).

## Discussion

The main findings from this study are: (1) the diurnal rise in intra‐hippocampal CORT levels in wild‐type mice increases with ageing; (2) 11β‐HSD1 deficiency substantially diminished this diurnal rise in intra‐hippocampal CORT, but not the diurnal rise in plasma CORT; (3) a swim stress during the diurnal hippocampal nadir caused a greater rise in intra‐hippocampal CORT levels in age‐matched wild‐type than 11β‐HSD1^−/−^ mice; (4) basal intra‐hippocampal CORT levels are unaffected by 11β‐HSD1 deficiency in young or old mice. Together, these data indicate a potent and dynamic control of CORT levels by 11β‐HSD1 in the dorsal hippocampus under diurnal peak and stress conditions without altering basal levels.

Under quiescent conditions, intra‐hippocampal CORT levels in wild‐type mice showed a diurnal pattern of variation with a nocturnal peak similar to that described in rats (Droste *et al*., 2009). It is notable that this peak was not only greater in magnitude but also lasted longer in aged wild‐type mice. Intriguingly, the intra‐hippocampal CORT peak was not seen in either young or aged 11β‐HSD1^−/−^ mice despite evidence of a normal rhythm in plasma CORT levels. However, although the peak increase in intra‐hippocampal CORT levels was not notable, a small diurnal rise was observed in 11β‐HSD1^−/−^ mice. The modest increases that were observed in 11β‐HSD1^−/−^ mice hippocampus during the dark phase probably reflect the ~5% of free CORT (i.e. not bound to CBG) entering the brain from the peripheral evening rise in CORT. In wild‐type mice, the substantially greater diurnal rise in intra‐hippocampal CORT levels is due to enhanced local regeneration of CORT from 11‐dehydrocorticosterone by 11β‐HSD1. Because 11‐dehydrocorticosterone (derived from 11β‐HSD2 metabolism of peripheral CORT) binds poorly to CBG, most of the circulating levels are free to enter the brain to be reactivated by 11β‐HSD1, thus making a significant contribution to total hippocampal CORT levels in wild‐type mice. Whether 11β‐HSD1 activity is diurnally regulated in the mouse hippocampus is not known, but glucocorticoids have themselves been shown to upregulate the transcription of 11β‐HSD1 (Sai *et al*., 2008). Taken together, these data suggest that the hippocampus is exposed cumulatively to higher levels of CORT in ageing, and that this higher level is in part due to CORT generated locally by 11β‐HSD1. Given the known deleterious effects of glucocorticoids, the sustained exposure of hippocampal GRs to high CORT in aged wild‐type mice may increase the vulnerability of the aged hippocampus to potential structural changes that underlie memory impairments (Nichols *et al*., 2001). Whether 11β‐HSD1 activity is the sole cause of age‐dependent changes in hippocampal GR activation or a constant modulatory component throughout life is not clear.

Studies of ageing in both humans and rodents have revealed marked alterations in the diurnal and/or circadian rhythms in plasma glucocorticoids, showing an increased basal activity and flattened diurnal amplitude of the HPA system (Deuschle *et al*., 1997; Spiga *et al*., 2014). Our data showing increased basal morning plasma CORT levels in the aged mice with no effect of 11β‐HSD1 deficiency confirm our previous findings (Yau *et al*., 2007), and are consistent with increased HPA activity. Given that a disrupted rhythm of plasma CORT is thought to be a hallmark of ageing, it would be of interest to determine whether this accompanies the intact diurnal cycle of intra‐hippocampal CORT levels observed in our aged mice. Ideally, cyclical changes in brain and plasma CORT levels should be measured simultaneously in the freely behaving animals by brain microdialysis and blood sampling (via jugular vein cannula) as described in rats (Droste *et al*., 2008). However, this is not technically feasible in mice.

Loss of local regeneration of CORT at sites where 11β‐HSD1 is highly expressed, notably in the hippocampus (Lakshmi *et al*., 1991; Roland *et al*., 1995) and paraventricular nucleus of the hypothalamus (PVN; Seckl, 1997) would be anticipated to dampen HPA feedback and increase plasma CORT levels in 11β‐HSD1^−/−^ mice. Indeed, swim stress induced a rise in intra‐hippocampal CORT levels in young and aged 11β‐HSD1^−/−^ mice, which was significantly lower than in wild‐type mice. However, just as diurnal variations in plasma CORT levels were similar in wild‐type and 11β‐HSD1^−/−^ mice, stress‐induced changes in plasma CORT were also not affected by genotype. This paradox is explained by adaptive responses in the adrenal gland, which has increased steroidogenic capacity in 11β‐HSD1^−/−^ mice to compensate for reduced regeneration of active glucocorticoids by the liver and other peripheral tissues. It is notable that hyperactivity of the adrenal gland in 11β‐HSD1^−/−^ mice is dependent on genetic variations in the hippocampus and PVN between mouse strains (Harris *et al*., 2001; Carter *et al*., 2009), and is rescued by liver‐specific transgenic expression of 11β‐HSD1 (Paterson *et al*., 2007).

Hippocampal tissue CORT levels, measured *ex vivo* from mice culled in the morning, are lower in aged 11β‐HSD1^−/−^ mice than aged wild‐type controls (Sooy *et al*., 2010; Yau *et al*., 2011). This contrasts with basal intra‐hippocampal CORT levels measured in dialysates from freely behaving mice, which were similar in wild type and 11β‐HSD1^−/−^ mice for much of the day (only increased in the evening in wild‐type mice). It is possible that the higher tissue CORT levels in aged wild‐type controls are due to HPA axis activation caused by handling prior to the cull. Indeed, just 10 min exposure to a novel environment has been shown to increase hippocampal CORT levels in freely moving C57BL/6 mice (Thoeringer *et al*., 2007). Similarly, exploration of the Y‐maze caused a greater rise in intra‐hippocampal CORT levels in aged wild‐type than 11β‐HSD1^−/−^ mice (Yau *et al*., 2015). Genotypic differences in intra‐hippocampal CORT levels were also evident following swim stress, with a greater rise in both young and aged wild‐type than in age‐matched 11β‐HSD1^−/−^ mice. This supports our hypothesis that 11β‐HSD1 activity in the brain *in vivo* is regulated in a dynamic manner with increases prompted by HPA axis activation. The subsequent increase in substrate availability for 11β‐HSD1, 11‐dehydrocorticosterone (via 11β‐HSD2 metabolism of peripheral CORT) may underlie the rapid rise in intra‐hippocampal CORT levels in wild‐type mice during the diurnal rise and following stress.

In conclusion, local regeneration of active glucocorticoids by 11β‐HSD1 in the dorsal hippocampus is dynamically regulated by environmental changes that activate the HPA axis, such as stress and diurnal cues (lights off), and that, with aging, the regenerative component is even greater perhaps reflecting age‐associated increases in 11β‐HSD1 expression in the hippocampus (Holmes *et al*., 2010). The stress‐induced rise in CORT generated by 11β‐HSD1 in the hippocampus may in part cause memory impairments in both young and aged mice (Yau *et al*., 2015). Selective 11β‐HSD1 inhibitors, shown to attenuate age‐related memory impairments in aged mice (Sooy *et al*., 2010; Yau *et al*., 2015), could be beneficial in the treatment of stress‐related cognitive disorders.
